# Efficient Electron Hopping Transport through Azurin-Based Junctions

**DOI:** 10.1021/acs.jpclett.3c02702

**Published:** 2023-12-07

**Authors:** Carlos Roldán-Piñero, Carlos Romero-Muñiz, Ismael Díez-Pérez, J. G. Vilhena, Rubén Pérez, Juan Carlos Cuevas, Linda A. Zotti

**Affiliations:** † Departamento de Física Teórica de la Materia Condensada, Universidad Autónoma de Madrid, E-28049 Madrid, Spain; ‡ Departamento de Física de la Materia Condensada, Universidad de Sevilla, PO Box 1065, 41080 Sevilla, Spain; ¶ Department of Chemistry, Faculty of Natural & Mathematical Sciences, King’s College London, Britannia House, 7 Trinity Street, London SE1 1DB, U.K.; § Condensed Matter Physics Center (IFIMAC), Universidad Autónoma de Madrid, E-28049 Madrid, Spain

## Abstract

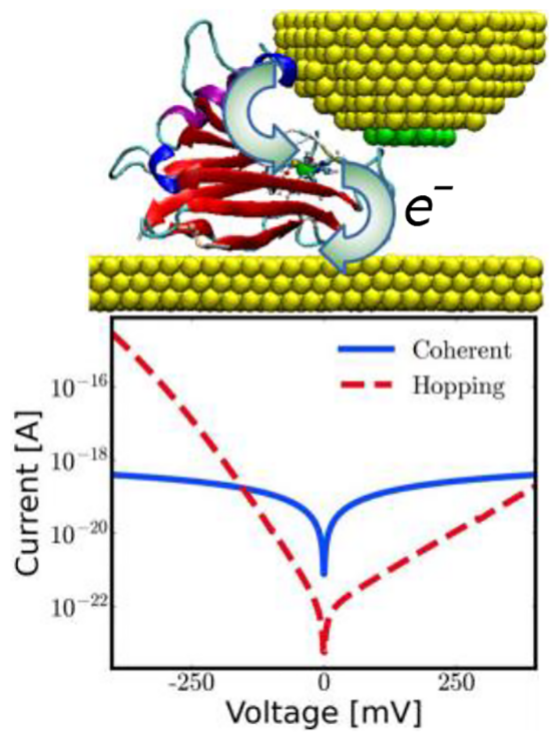

We conducted a theoretical study of electron transport through junctions of the blue-copper azurin from *Pseudomonas aeruginosa*. We found that single-site hopping can lead to either higher or lower current values compared to fully coherent transport. This depends on the structural details of the junctions as well as the alignment of the protein orbitals. Moreover, we show how the asymmetry of the *IV* curves can be affected by the position of the tip in the junction and that, under specific conditions, such a hopping mechanism is consistent with a fairly low temperature dependence of the current. Finally, we show that increasing the number of hopping sites leads to higher hopping currents. Our findings, from fully quantum calculations, provide deep insight to help guide the interpretation of experimental *IV* curves on highly complex systems.

The field of protein electronics has flourished remarkably over the past decade.^1^ The interest in these systems is mainly triggered by the highly efficient charge-transfer that proteins can exhibit over long distances,^2,3^ besides their role in extremely important processes such as in the respiratory and photosynthetic chains.^4,5^ However, experimental advances have also enabled the study of electron transport through proteins incorporated in solid-state junctions.^6,7^ This has paved the way for the development of future electrical devices based on proteins as active elements, as well as their use in sensors and biocompatible devices.^8^ In addition, interesting mechanical, self-assembly, chemical recognition, and optoelectronic properties have been revealed which could be exploited in the development of such new-generation devices. Among various types of proteins studied in the field, the blue-copper azurin from *Pseudomonas aeruginosa* has been analyzed quite extensively.^9−13^ These studies brought to light several surprising electron-transport properties: these include the possibility of inducing drastic changes in the gate-voltage dependence via a single amino acid mutation,^14^ the lack of temperature dependence down to 4 K^12,15,16^ (which was interpreted as an indication of coherent tunneling), and the considerably high conductance values (up to 10^–5^*G*_0_ in single-protein experiments^17^) observed despite its large size.^17^ Nevertheless, to date, the exact nature of the transport mechanism through this protein is still the subject of a long-standing debate;^15,18^ several types of processes such as 2-step tunneling,^14^ fully coherent transport,^19^ and carrier-cascade^20^ have been proposed. Recently, some of us have shown that fully coherent tunneling through a single-azurin junction would lead to extremely low conductance values as compared to those observed in experiments.^18^ This was deduced from a density functional theory (DFT)-based study, which involved a high number of geometrical structures obtained via molecular dynamics simulations (MD), reproducing a broad range of structures likely formed in STM experiments. A subsequent study of ours revealed that, within the same transport mechanism, the role of the central metallic ion would not be as relevant as expected compared to other residues of the protein.^21^ These findings have cast doubts on the actual role of fully coherent transport with respect to other types of transport processes (such as sequential tunneling, for instance), which were otherwise suggested.^14^ Clarifying such a basic issue is paramount in the prospect of developing protein-based electronics and optimizing the performance of any electrical device incorporating these kinds of systems. Therefore, in order to shed light on this puzzle, we hereby extend our investigation on azurin-based junctions to the analysis of an incoherent type of electron transport, which until now was only tackled by means of simple models^15^ (which do not include the complexity of the whole electronic structure of these systems). In particular, we have analyzed a hopping process through either the Cu ion or a histidine residue which was previously found to be relevant in the tunneling process.^21^ We will show that the hopping currents can be higher or lower than those obtained in a fully coherent transport depending on structural details of the junctions such as the tip–protein contact. These also affect the asymmetries in the *IV* curves, depending on the different coupling established with the electrodes.

More specifically, we have computed current–voltage (*IV*) curves based on an incoherent-transport (hopping) model for gold–azurin–gold junctions, such as those displayed in Figure 1a. The geometrical structures were obtained via MD simulations, the details of which were reported in previous works.^18,22^ There, the study of their electronic-structure properties is also presented. They were extracted via a fully quantum approach based on DFT calculations which were performed by using the code OpenMX.^23,24^ We started our investigation by focusing on the Cu ion, which previous literature^14,15,25^ indicated as the main player in the electron transport through this protein. In the model employed in this study, an electron travels between a substrate and a tip via hopping on the Cu ion (which is located in the central area of the protein), as depicted in Figure 1b. The black arrows indicate the transfer rates for each of the two steps in each direction. In the following, we will refer to the substrate and tip as the left and right electrode, respectively.

**Figure 1:**
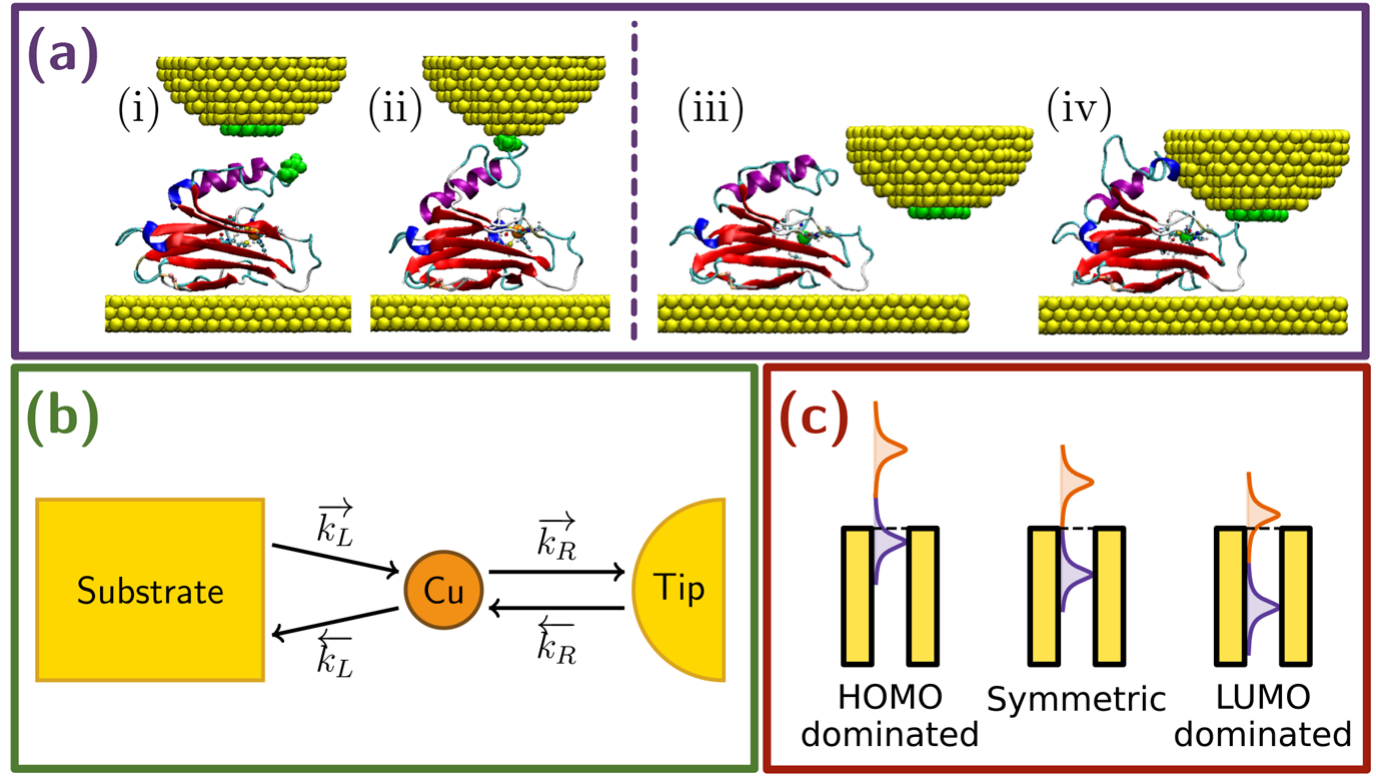
(a) Initial (i, iii) and final (ii, iv) geometries for the MD simulations mimicking junction formation through blinking (left) and side-indentation (right), respectively. (b) Schematic representation of the electron-transport mechanism taking place in a metal–azurin–metal junction via hopping through the Cu ion. (c) Schematic representation of the three cases considered for the level alignments.

In the framework of the Marcus theory,^26−28^ for a given bias voltage *V* applied between left and right electrode, the transfer rates between the leads and the Cu ion can be calculated as
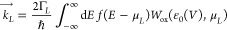
1
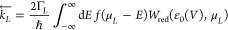
2
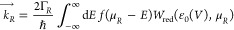
3
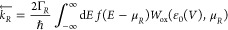
4where *f*(*E*) are the Fermi distribution functions, while μ_*L*/*R*_ is the voltage-induced displacement of the chemical potential of either the left or the right metallic electrode, calculated as
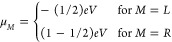
5In our model, the Fermi level of the left (right) electrode is shifted downward (upward) in energy for positive bias, while it is shifted in the opposite directions for negative bias. *W*_ox/red_ are distribution functions given by
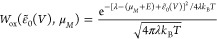
6
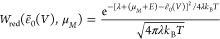
7where λ is the reorganization energy of the hopping center and  is the position of the level, which changes as a function of the bias voltage (see below for further details). Here, Γ_*L*/*R*_ is the level broadening associated with the coupling with the left or right electrode. They were calculated as
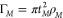
8where ρ_*M*_ is the average density of states per atom in the left or right electrode while *t*_*M*_ can be interpreted as a through-bridge tunneling matrix element.^27^ All quantities (except for the reorganization energy) were obtained from the output of the DFT calculations performed on the whole metal–protein–metal junctions. In particular, the evaluation of *t*_*M*_ required two distinct postprocessing calculations for the left and right metal–protein interfaces. In our procedure, the total Hamiltonian *H* is first divided into blocks as follows:
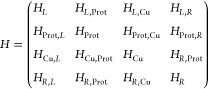
9where *H*_*L*_, *H*_Cu_, and *H*_*R*_ are the blocks relative to the left electrode, the Cu ion, and the right electrode, respectively, while *H*_Prot_ is the block corresponding to the protein deprived of the Cu orbitals. The off-diagonal terms correspond to the interactions among these four main components. Within the limit of validity of perturbation theory, *t*_*L*_ is then given by *H*_*L*,Prot_*g*(*E*)*H*_Prot,Cu_, whereas *t*_*R*_ is given by *H*_Cu,Prot_*g*(*E*)*H*_Prot,*R*_. Here, *g*(*E*) is Green’s function corresponding to the protein deprived of the Cu-ion, which acts as the bridge in both steps (metal–Cu and Cu–metal). Obviously, for each process, the residues in the protein which are spatially located far away from the direct path between Cu and the electrode do not contribute significantly. More specifically, the Green’s function *g*(*E*) is calculated as
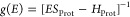
10*S*_Prot_ being the corresponding block of the overlap matrix. The dependence of the uncorrected position of the level ε_0_ on the coupling with the electrodes and the bias voltage was taken into account by calculating it as follows:
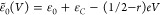
11Here, ε_*C*_ is a correction that we apply to the molecular orbitals of the protein in order to address the inaccuracy induced in the energy-level alignment by the use of the DFT-GGA approach. This was achieved by means of a scissor-like correction^29^ which results in an increase of the HOMO–LUMO gap.

The quantity *r* serves the purpose of addressing the energy displacement of the level ε_0_ due to the coupling with the electrodes.
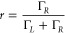
12

Finally, the current was computed as^15,27^
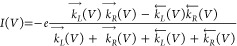
13

In order to account for a possible role of more than one hopping site, we also explored the use of a three-site model, in which the central site is the metal ion whereas the other two are the two portions of protein comprised between the ion and the electrode on each side. In this case, the analytical expression of the current becomes more cumbersome. The details about the extraction of the current values in this case can be found in the SI. For the sake of comparison, for selected cases, we compared the current values with those obtained within a fully coherent mechanism. In this case, the current is given by the Landauer formula
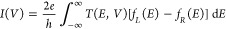
14where *f*_*L*/*R*_(*E*) are the Fermi functions of left and right electrodes. The transmission *T*(*E*, *V*) has been computed by Green’s function techniques as described in ref (18) upon applying the same gap correction as for the hopping model (see below for details).

We now turn to analysis of the computational results. We have considered single-protein junctions that mimic structures likely to be obtained with STM-based techniques. In the present study, these include two main sets of geometries: (i) junctions obtained by simulating the blinking technique and (ii) junctions obtained by simulating a lateral indentation (Figure 1a). In the former, the tip is positioned in the proximity of the protein, avoiding physical contact. However, thermal fluctuations induce continuous attachment and detachment from the tip. Jumps in the current signal are detected whenever chemical bond is established.^14,30−33^ In the latter, the tip approaches the protein sideways, while it is kept at a fixed distance from the surface. As mentioned before, the study of the mechanical and electronic properties of these structures (obtained by MD and uncorrected DFT-GGA) was reported in previous works.^18,22^

In the present study, we have considered three possible scenarios for the zero-bias alignment of the protein with respect to the Fermi level of the electrodes (see Figure 1c): the Fermi level lying in the middle of the HOMO–LUMO gap (i) and either the HOMO (ii) or the LUMO (ii) being very close to the Fermi level. For the sake of simplicity, we will refer to these three cases as symmetric, HOMO-dominated, and LUMO-dominated, respectively. For all of them, based on previous literature,^34^ the size of the HOMO–LUMO gap was increased by 1 eV with respect to the GGA value. The reason for considering these three representative cases lies in the conflicting information reported in the literature regarding the level alignment for this system.^14,21,34−37^ For small organic molecules, the position of the frontier orbitals can be extracted via differences of total energies of neutral and charges states.^29,38^ DFT calculations of charged states of a complex system such as the entire azurin protein (almost 2000 atoms), however, are not straightforward and may easily lead to incorrect results. Therefore, we prefer to turn to analyzing these three clear-cut situations so as to cover all possibilities. Note that in any case they would also correspond to the different scenarios induced by applying different gate-voltage values.

A key ingredient in Marcus theory is given by the reorganization energy. Over the years, various values have been reported for the blue-copper azurin.^34,39−41^ For the present work, we employed a value of 0.5 eV. Nevertheless, in the SI we show examples of curves obtained with different reorganization-energy values.

Previous literature indicated the Cu ion as the main player in electron transport through azurin junctions.^14,15,25^ In the case of the HOMO-dominated and the symmetric case, this seems to be plausible given the presence of Cu orbitals among the highest occupied energy levels of the protein.^18,37^ Thus, the Cu ion was chosen as the hopping site. For the LUMO-dominated case, however, the same choice does not appear as suitable, since the Cu unoccupied states lie well above the Fermi level. Indeed, our DFT calculations revealed that in these structures, the LUMO is not localized on the Cu ion but rather on the residue HIS35, which is positioned between the Cu ion and the surface. Consequently, for the LUMO-dominated case, this residue was chosen as the hopping site. For this, the same reorganization-energy value as that for Cu was chosen (curves obtained with different values are shown in the SI). It should be noted that the HIS35 residue was already found to be particularly relevant within a fully coherent transport.^21^

Figure 2 displays the *I*(*V*) curves obtained for the blinking (a) and side indentation (b). For each case, we show three sets of curves, corresponding to three representative time frames for the blinking and three different tip–protein distance values for the lateral indentation. Such a distance was calculated between the Cu ion and the center of the lowest tip layer. The majority of the curves shown in this figure exhibits some degree of asymmetry, their values at positive voltage being either higher or lower than in the negative range depending on the coupling with the leads. It is well-known, indeed,^26^ that asymmetries in *IV* curves mostly originate from geometrical asymmetries in the junction. In fact, differences in the coupling at the molecule–metal interface can affect the voltage profile by inducing an energy shift in a molecular orbital in the same direction as the chemical potential of the electrode with which it is more strongly coupled (as described, in our model, by eq 5). The curves in Figure 2, in particular, seem to reflect the differences in the tip–protein contact between the two schemes. In the final steps of the lateral indentation, the tip becomes significantly close to the Cu complex, making the Cu–lead coupling on that side larger than that on the opposite side. Conversely, in the blinking process, a separation between the α helix and β barrel is induced by formation of the metal–protein contact,^22^ making the Cu–tip coupling weaker than on the surface side. In the symmetric scenario considered in our model, for instance, in the case for which the coupling between the Cu ion and the tip becomes more relevant than the Cu–surface coupling, values at positive bias become higher than those at negative bias. The opposite applies to the reversed situation. This is particularly visible in the symmetric case for the lateral indentation, where the Cu–tip coupling increases as the tip approaches the protein, reversing the asymmetry (blue curve vs red and green curves). This does not happen for the blinking case, since there the Cu–tip coupling is much lower than in the side indentation. Interestingly, the same reasoning does not seem to apply to the HOMO- and LUMO-dominated cases, where this effect seems to be counteracted by the proximity of the orbital to the Fermi level. An in-depth discussion of this issue may be found in the SI. It should be noted that such a detailed analysis of the asymmetries is possible thanks to having obtained the whole electronic structure of the entire metal–protein–metal junction at the DFT level and to having calculated the coupling elements by means of the perturbation-theory approach described above. It is also worth mentioning that asymmetries in the *IV* curves were indeed observed in some of the experimental measurements on this protein (see, for instance, Figure S3 of ref (42)).

**Figure 2:**
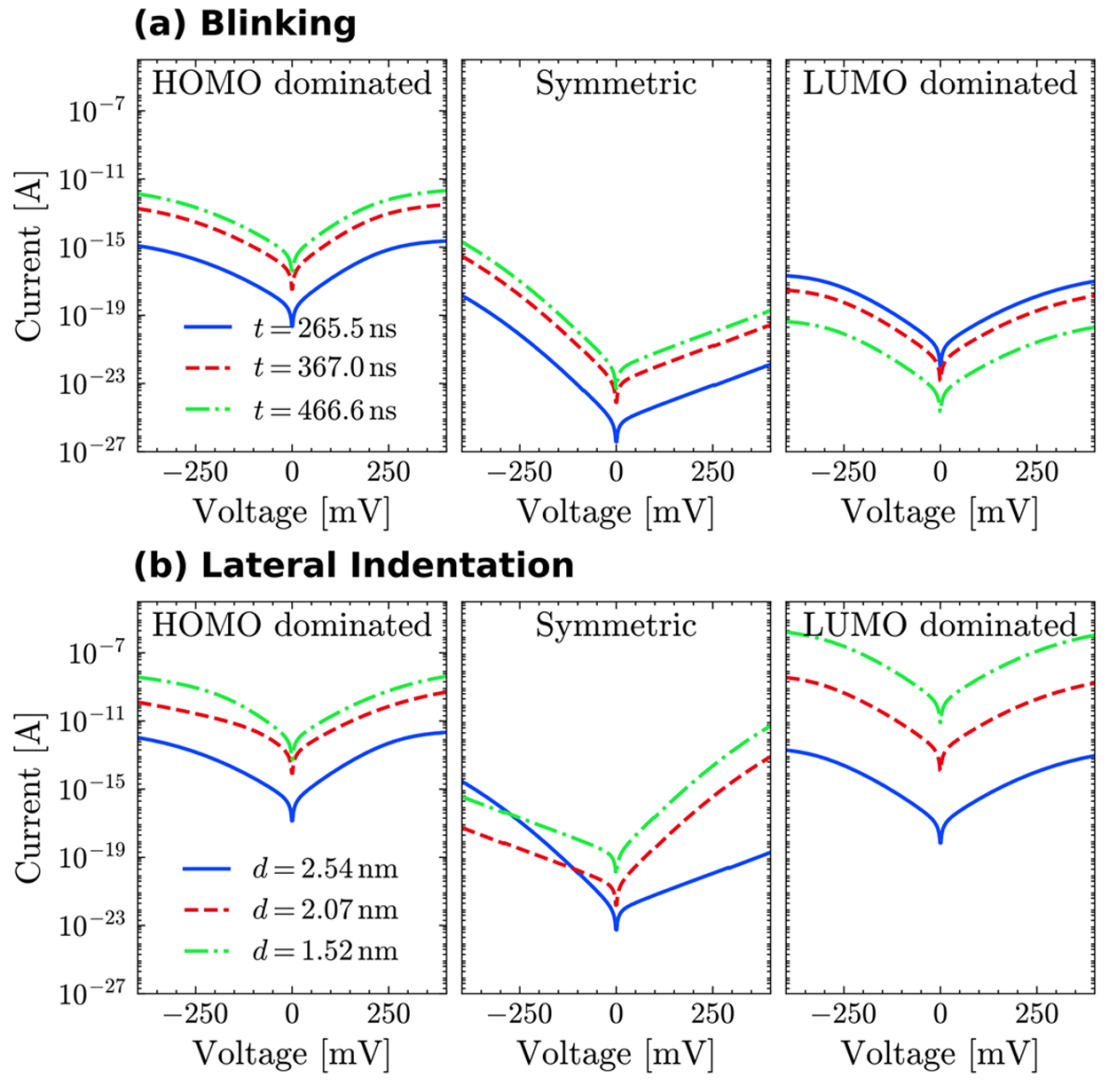
Hopping currents through one site in the blinking (a) and lateral (b) scheme for the HOMO-dominated, symmetric, and LUMO-dominated cases.

Overall, the lateral indentation yields higher current values than the blinking. It should be noted that the same was observed for the fully coherent transport through the same system and is mainly due to the shorter tip–surface distance in the side-indentation junctions.^18^ For the blinking method, we find that the highest current values are obtained within the HOMO-dominated scheme. For the lateral indentation, instead, the highest values are obtained for the LUMO dominated case. This is probably due to the strong coupling between the surface and residue HIS35, which is the hopping site in this case. As this residue is positioned between the Cu ion and the surface, however, this effect fades away in the blinking scenario, where the larger HIS35–tip distance leads to a decrease in the current.

We now turn to analyzing the temperature dependence of the current, which has been the subject of several studies focused on the azurin.^9^ In Figure 3a, we report, as an example, the current as a function of the inverse of the temperature for a range between 10 and 500 K for two selected cases (*t* = 466.6 ns for the blinking and *d* = 2.54 nm for the side indentation), fitted to the following exponential equation:
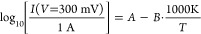
15

**Figure 3:**
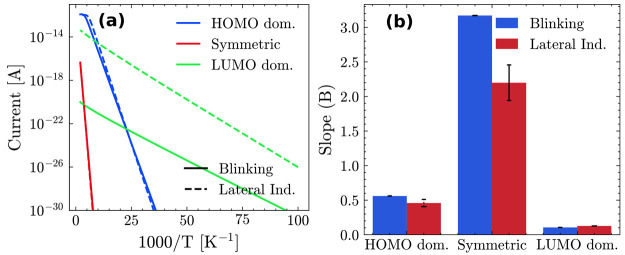
(a) Temperature dependence of the one-site hopping currents obtained for blinking (at *t* = 466.6 ns) and for the lateral indentation (for *d* = 2.54 nm). (b) Barplot with the values of the fitting parameter *B* of eq 15 for all cases considered.

This fit was performed on all of the MD simulation frames. In Figure 3b we report a bar plot with the average slope values obtained for both sets. In particular, we observe a weak time dependence (*B* = 0.11–0.16) for the LUMO-pinning case in the side indentation. It should be noted that the slope increases with the value of the reorganization energy assigned to HIS35 (see SI).

We now turn to comparing the hopping *I*(*V*) curves with those obtained for a fully coherent transport (in the spirit of the Landauer formalism, Figure 4). For each method of contact formation, we considered three different MD frames for the three level-alignment situations.

**Figure 4:**
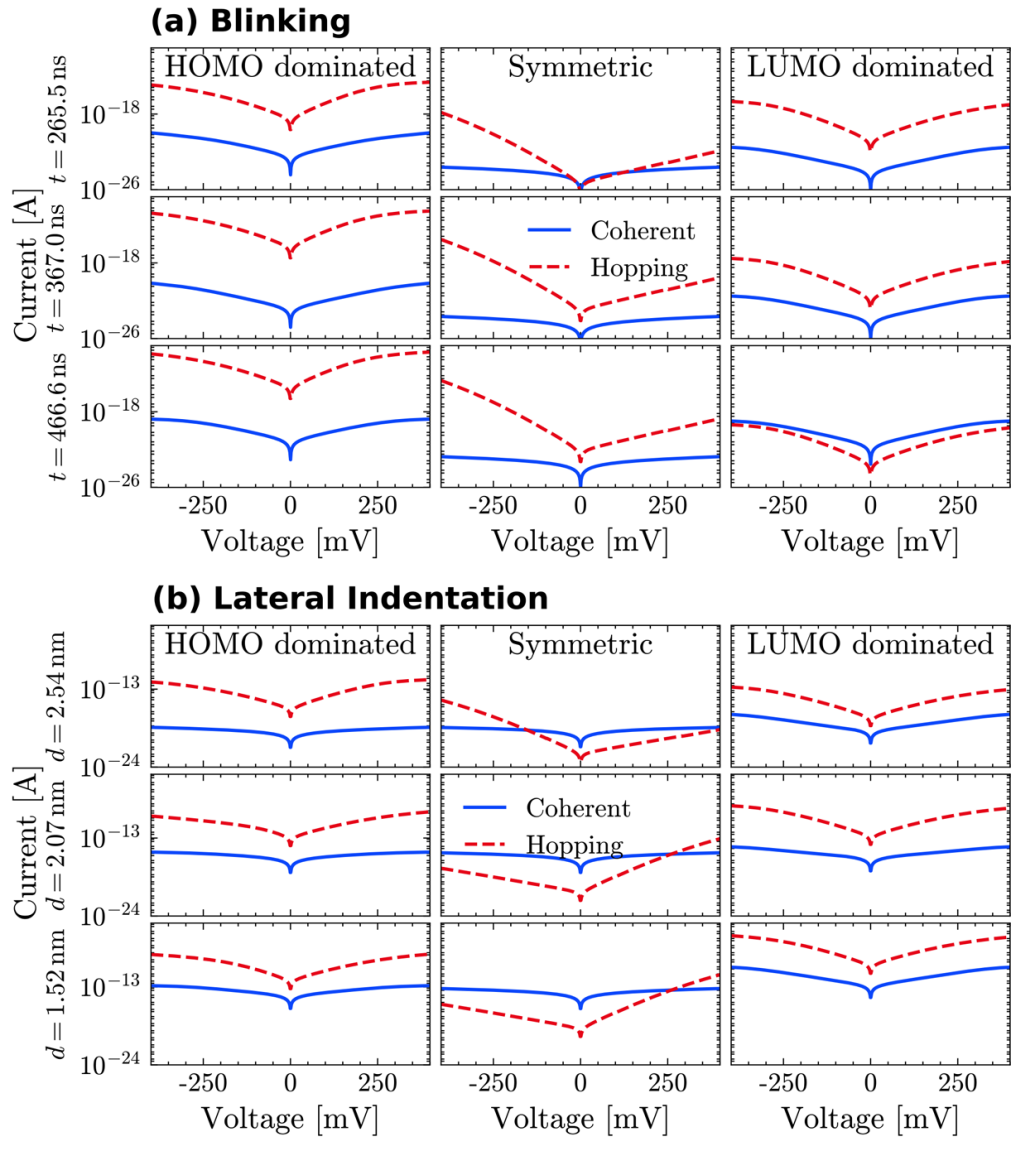
One-site hopping and coherent-transport *IV* curves for three selected MD time frames of the blinking (a) and the lateral indentation (b) simulations in the HOMO-dominated, symmetric, and LUMO-dominated cases.

In most cases, the current calculated for a hopping mechanism appears to be higher than the corresponding one obtained within a fully coherent transport. This is not the case, however, for the lateral indentation in the symmetric scheme, especially at a short tip–protein distance. In particular, we observe that the coherent-transport currents for the HOMO-dominated case are similar to those from their symmetric counterparts. This is because the HOMO resonance is extremely sharp^18^ (as compared, for instance, to the LUMO resonance^21^). Overall, these results seem to suggest that within hopping through a single site, preference over one transport mechanism or the other may depend on both the specific gate-voltage applied and the geometrical position of the tip with respect to the protein.

Finally, we stress that one should not rule out the possibility of more hopping sites taking place in the transport process. Previous works of ours^22,37^ highlighted the presence of several states lying below the HOMO and energetically very close to it. These states originate from moieties other than the Cu ion, and they are located all over the peripheral area of the protein structure. In order to obtain an approximate estimate of their possible contribution in electron transport, we built a model in which each hopping site comprises several of these residues. In Figure 5, we show the current curves for a time frame of the lateral-indentation simulation obtained for hopping through three sites in the symmetric scheme. More specifically, the first site includes the residues ASP11, HIS46, ASP93, GLU104, and GLU106, which are spatially located between the surface and Cu. The second hopping site is the Cu ion. The third site includes the residues ASP55, ASP69, ASP71, ASP76, CYS112, and HIS117, which are comprised between the Cu ion and the tip. These residues were chosen as they all contribute to states very close to the Fermi level (within a range of 0.2 eV). We have assigned two different reorganization energy λ_*L*_ and λ_*R*_ to the first and third site, respectively. It is possible to observe that regardless of the values of these two parameters, the current would be significantly higher than assuming hopping through the Cu ion only. We refer the reader to the SI for a more detailed analysis of the role of the number of hopping sites.

**Figure 5:**
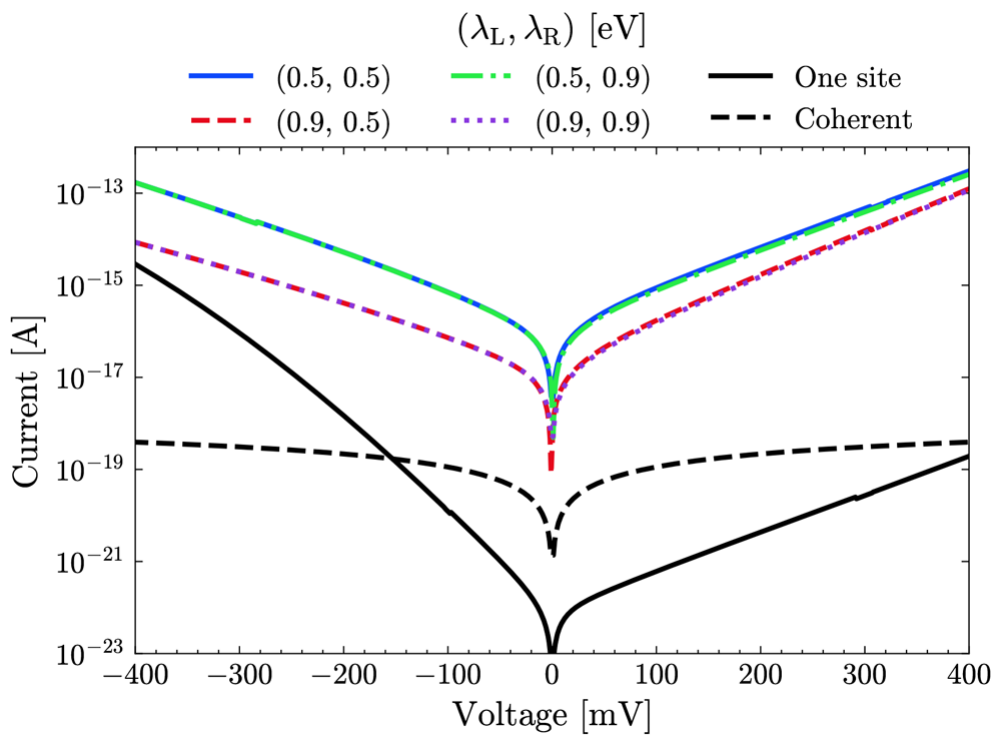
Comparison between the current obtained for one-site and three-site hopping for different values of the reorganization energies of the two sets of residues forming the first and third sites (λ_*L*_ and λ_*R*_, respectively). These specific data were extracted from the lateral-indentation scheme for *d* = 2.54 nm.

A quantitative comparison to experimental results is not straightforward. It would require a systematic study of experimental *I*(*V*)s as a function of the tip-to-surface gap separation. While this is experimentally reachable, this study implies a large block of experimental work and is beyond the scope of the current study. However, we observe that the current values reported in the literature for single-azurin experiments (around 1/2 nA^42^) would not be achieved in the symmetric scheme but rather in a situation in which one of the two frontier orbitals lies closer to the Fermi level.

In summary, we have studied electron transport through a metal–protein–metal junction based on a blue-copper azurin. By adopting a procedure based on fully quantum calculations, we have taken into account the whole atomic and electronic structure of the entire junction. We found that the asymmetry of the *I*(*V*) curves is strongly affected by the specific position of the tip in the junction. We also found that within a one-site hopping framework, hopping currents can be higher or lower than the coherent-transport currents depending on the energy alignment of the protein orbitals with respect to the Fermi level. Higher hopping currents can be obtained, however, by considering more than one stepping site. Finally, we addressed the very low temperature dependence of the conductance, which was previously observed in several experiments. We have shown that this hopping framework allows for such independence under certain conditions related to the energy alignment of the protein orbitals and to the reorganization energies of the hopping sites. To a certain extent, by showing that drastic changes can be induced in the transport mechanism by the various factors we have analyzed, our results provide an explanation for the presence of conflicting conclusions drawn in the previous literature. In view of the high complexity of these systems, we believe that our results provide guidance for the interpretation of the experimental results.

## Supporting Information Available

Theoretical details for an N-site hopping model (S1); the role of the protein–electrode coupling in the asymmetry of the *IV* curves; (S2) dependency of hopping rates on the bias voltage (S3); dependency of hopping currents on reorganization-energy values (S4); and temperature dependence for different reorganization-energy values (S5) (PDF)
