# Supplementary material for: Efficient Electron Hopping Transport through Azurin-Based Junctions
Source: J Phys Chem Lett. 2023 Dec 7;14(49):11242–9. doi: 10.1021/acs.jpclett.3c02702 (PMC10726356; doi:10.1021/acs.jpclett.3c02702)
Supplement: Supplementary file 1 — jz3c02702_si_001.pdf [file jz3c02702_si_001.pdf]

Efficient Electron Hopping Transport through Azurin-Based Junctions

Carlos Roldán-Piñero,[†] Carlos Romero-Muñiz,[‡] Ismael Díez-Pérez,[¶] J. G. Vilhena,[†,§] Rubén Pérez,[†,§] Juan Carlos Cuevas,[†,§] and Linda A. Zotti[*,†,§]

†*Departamento de Física Teórica de la Materia Condensada, Universidad Autónoma de Madrid, E-28049 Madrid, Spain*

‡*Departamento de Física de la Materia Condensada, Universidad de Sevilla, PO Box 1065, 41080 Sevilla, Spain*

¶*Department of Chemistry, Faculty of Natural & Mathematical Sciences, King's College London, Britannia House, 7 Trinity Street, London SE1 1DB, UK*

§*Condensed Matter Physics Center (IFIMAC), Universidad Autónoma de Madrid, E-28049 Madrid, Spain*

E-mail: linda.zotti@uam.es

Contents

S1 N-site hopping model **S3**

S2 Role of the protein-electrode coupling on the asymmetries of the IV curves **S6**

S3 Voltage dependence of the transfer rates **S8**

S4 Dependency of current on the reorganization energy **S9**

S5 Temperature dependence for different reorganization-energy values **S11**

S1 N-site hopping model

The general hopping model for a a chain of N sites connecting left and right electrode is described by the following set of equations, for each i site, with $i = 1, ..N$.

$$-(k_{i,i-1} + k_{i,i+1})P_i + k_{i-1,i}P_{i-1} + k_{i+1,i}P_{i+1} = \mathrm{d}P_i/\mathrm{d}t \tag{S1}$$

with $\sum_{i=1}^{N+1} P_i = 1$ and $P_0 = P_{N+1}$. The 0 and $N+1$ indexes indicate the left and right electrode, respectively. $k_{0,1}$, $k_{1,0}$, $k_{N,N+1}$ and $k_{N+1,N}$ correspond to $\overrightarrow{k_L}$, $\overleftarrow{k_L}$, $\overrightarrow{k_R}$ and $\overleftarrow{k_R}$ of equations (1) to (4) of the main text, respectively, while $k_{i,i+1}$ is given by

$$k_{i,i+1} = \frac{2\pi}{\hbar} \frac{1}{\sqrt{4\pi(\lambda_i + \lambda_{i+1})\mathrm{k_B}T}} \exp\left\{-\frac{[(\lambda_i + \lambda_{i+1}) + \tilde{\epsilon}_{i+1} - \tilde{\epsilon}_i]^2}{4(\lambda_i + \lambda_{i+1})\mathrm{k_B}T}\right\} . \tag{S2}$$

By setting the time derivatives to zero, the steady-state current evaluated at the $\{i,\ i+1\}$ interface may be computed as

$$I(V) = -e\left[k_{i,i+1}(V)P_i(V) - k_{i+1,i}(V)P_{i+1}(V)\right] . \tag{S3}$$

For $N = 1$, $I(V)$ takes the form of eq. (14) in the main text.

In order to shed light on the role of the number of hopping sites, we have extended the number of sites from three (as in the main text) to five. For that, we have divided both site 1 and site 3 of the 3-site model into two sites, according to the geometrical position within the protein. This gives rise to a 5-site chain, corresponding to the following redistribution:

- Site1: residues 104 and 106
- Site 2: residues 11,46 and 93
- Site 3: Cu ion
- Site 4: residues 112 and 117

- Site 5: residues 55, 69, 71 and 76

In Figure S1, the blue "Au-1-2-Cu-4-5-Au" curve corresponds to sequential hopping through all five sites. This model gives rise to lower current than in a 3-site hopping (black curve), showing that increasing the number of sites indefinitely does not result into a continue increase of current. These results indicate that an initial increase of hopping sites raises the current values thanks to an increased communication between the leads and the central Cu ion. However, a further increase of sites makes the flow less direct and coherent. The green, purple, yellow and light-blue curves corresponds to the cases in which, out of the five sites, we have removed one of them in each. Interestingly, we observe that the current drops when site 5 is removed. As this site corresponds to the residues which are closer to the tip (including residues 69, particularly responsible for the binding), these results seem to suggest that, in both the 3-site model and the 5-site model, the largest contribution to the current increase is provided by the residues which are closer to the tip.

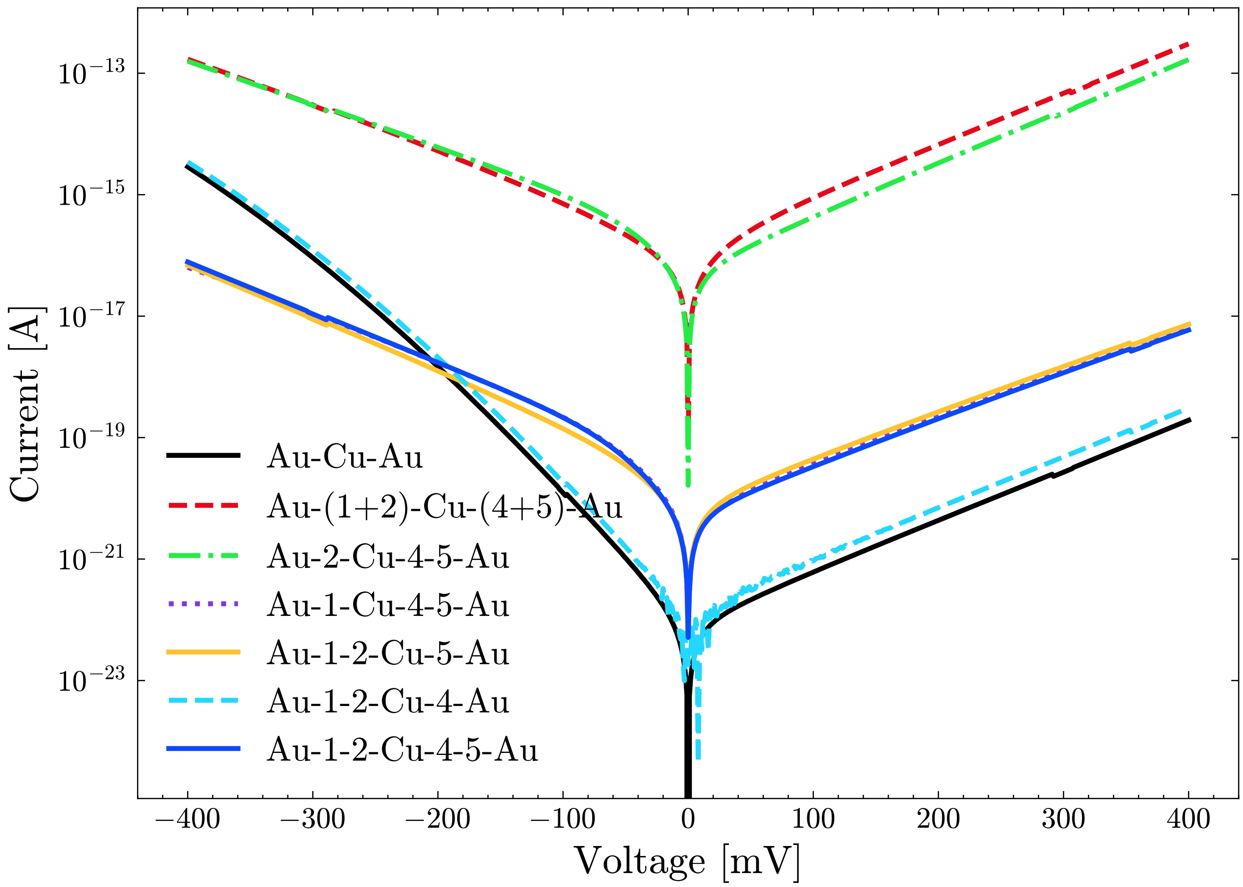

Figure S1: IV curves for a five-site hopping model, including all five sites (dark solid blue) or removing one of them (green, purple, yellow and light blue curves). Results are compared with those from the 1-site (black curve) and 3-site (dashed red curve) models.

S2 Role of the protein-electrode coupling on the asymmetries of the IV curves

In this section we provide a more quantitative explanation for the asymmetries observed in Fig. 4 of the main text by analyzing the three level-alignment cases separately.

In the symmetric case, both HOMO and LUMO are shifted far away from the Fermi level. Such a correction makes $W_{\mathrm{red}}\left(\varepsilon_0(V), \mu_L\right)$ and $W_{\mathrm{ox}}\left(\varepsilon_0(V), \mu_R\right)$ highly suppressed. Consequently, the current expression of eq. (14) of the main text can be approximated as:

$$I(V) \approx -e\left[\frac{\overrightarrow{k_R}(V)}{\overleftarrow{k_R}(V)} - \frac{\overleftarrow{k_L}(V)}{\overrightarrow{k_L}(V)}\right] . \tag{S4}$$

In the blinking scheme, the Cu ion is much closer to the substrate than to the tip. Consequently, the bottleneck for the current flow is represented by the Cu→ tip step. Thus, the easier this step is the higher the current. At negative voltages, the chemical potential of the substrate is shifted upwards, dragging the hopping site (the highest occupied Cu orbital) it is strongly coupled with. Conversely, the chemical potential of tip moves downwards. Overall, this reduces the energy distance between the hopping level and the chemical potential of the tip, facilitating the Cu-tip step ($\overrightarrow{k_R}$ increases), thus increasing the current . At positive voltage, the shifts of the chemical potentials of the electrodes are shifted, giving rise to lower currents. The same applies to the first frame of the lateral indentation (corresponding to large tip-protein distance). However, when the tip becomes closer to the protein (hence to the Cu hopping site), the situation is reversed since the bottleneck is, this time, rather given by the Cu-surface path, thus reversing the asymmetry in the curves.

In the HOMO-pinned case, the proximity of the HOMO to the Fermi level makes all the four hopping rates be relevant, reaching an approximately-constant value at higher voltages. This leads to a saturation of the current, in this range, at both biases and to a higher degree of symmetry of the curves.

In the LUMO-pinned case, the very-high coupling between the hopping site (HIS35) and the

substrate allows us to express the current as:

$$I(V) \approx -e \left[\frac{\overrightarrow{k_R}(V)}{\overleftarrow{k_L}(V)} - \frac{\overleftarrow{k_R}(V)}{\overrightarrow{k_L}(V)} \right] , \tag{S5}$$

and both ratios scale similarly. For both, in contrast to the symmetric case, the scaling of the denominator is similar to the numerator. Overall, this effect causes the saturation observed in the IV curves.

S3 Voltage dependence of the transfer rates

In this section, we show an example of how the four different hopping rates of eq.14 in the main text behave upon change in the bias voltage.

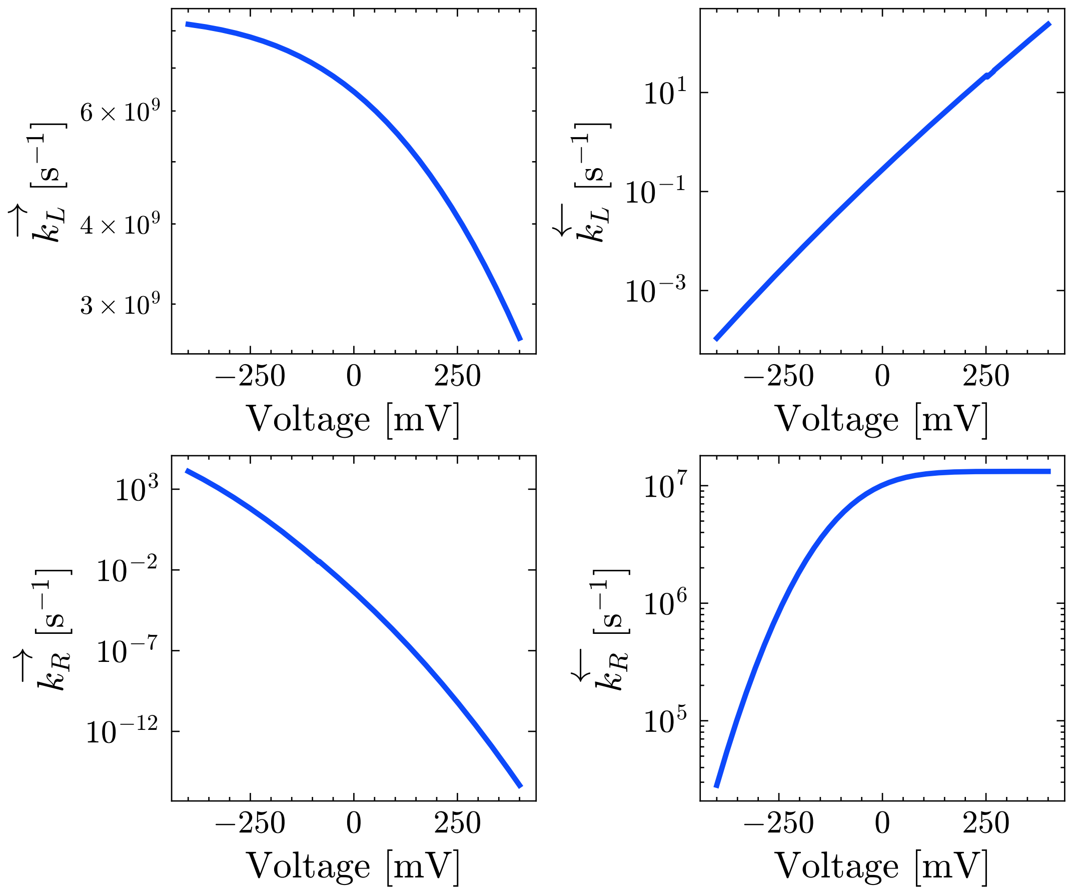

Figure S2: Values of $\overrightarrow{k_L}$ (upper left), $\overleftarrow{k_L}$ (upper right), $\overrightarrow{k_R}$ (lower left) and $\overleftarrow{k_R}$ (lower right) for hopping through the Cu ion in the blinking scheme for $t = 466.6\,\mathrm{ns}$ in the symmetric case.

S4 Dependency of current on the reorganization energy

In this section, we show examples of how the 1-site hopping current changes with the reorganization energy of the hopping site. In general, we observe that low reorganization energies lead to higher current values.

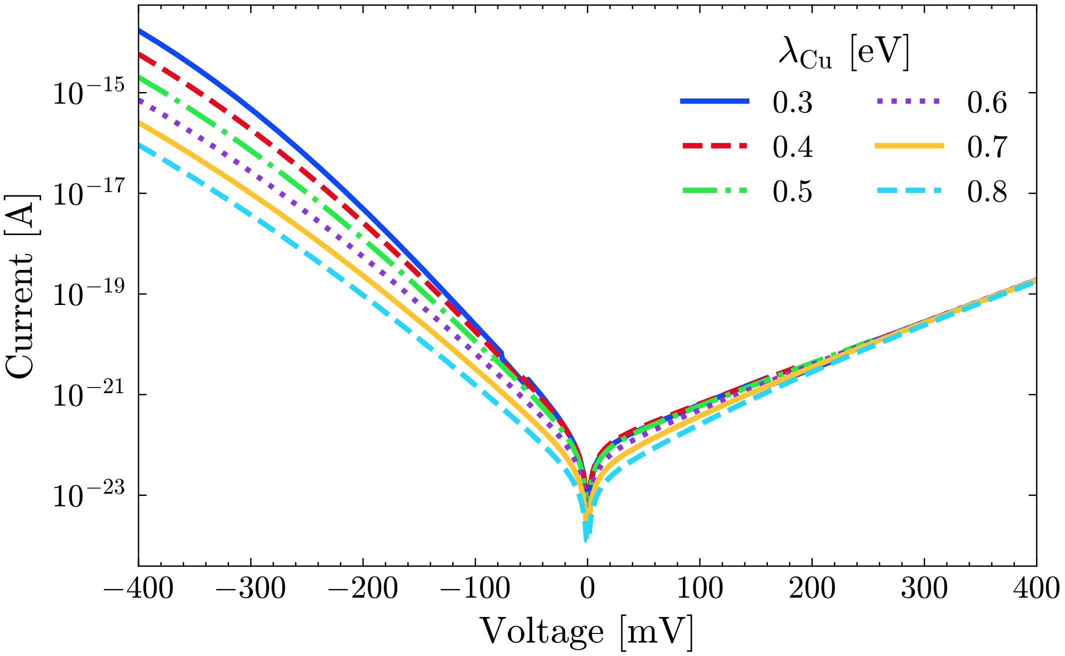

Figure S3: Dependency of the current with the reorganization energy of copper. Data were extracted from the blinking scheme at $t = 466.6\,\text{ns}$ in the symmetric case.

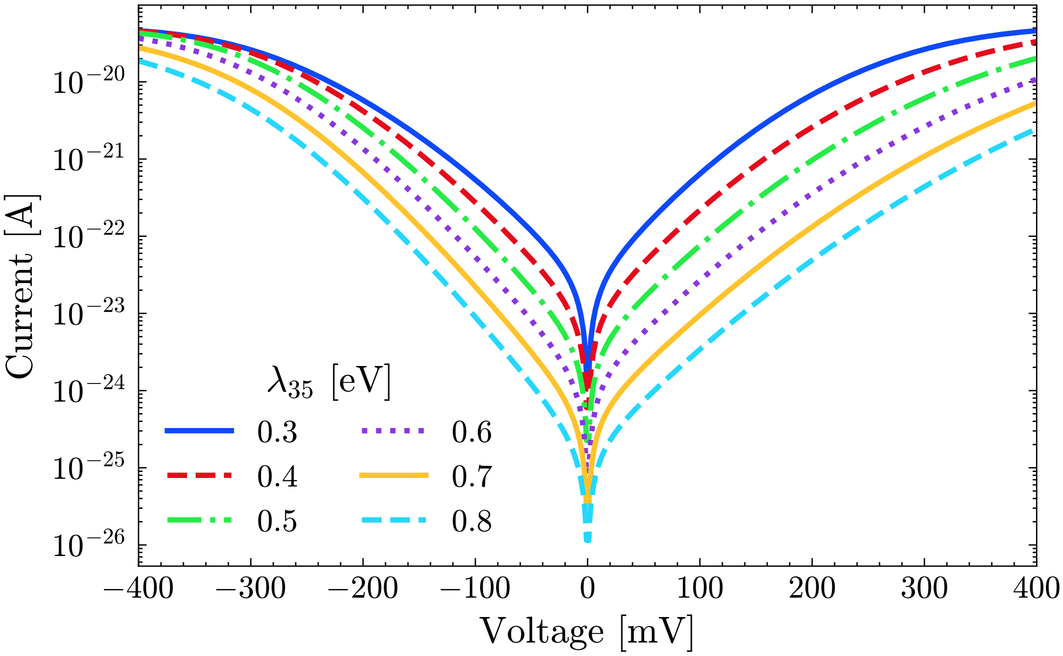

Figure S4: Dependency of the current on the reorganization energy of residue HIS35. Data were extracted from the blinking scheme at $t = 466.6\,\mathrm{ns}$ in the LUMO-pinned case.

S5 Temperature dependence for different reorganization-energy values

In the figure below, we show how the temperature-dependence of the hopping current through HIS35 is affected by the reorganization energy assigned to this residue. It can be observed that such a dependency becomes very low for low values of this energy.

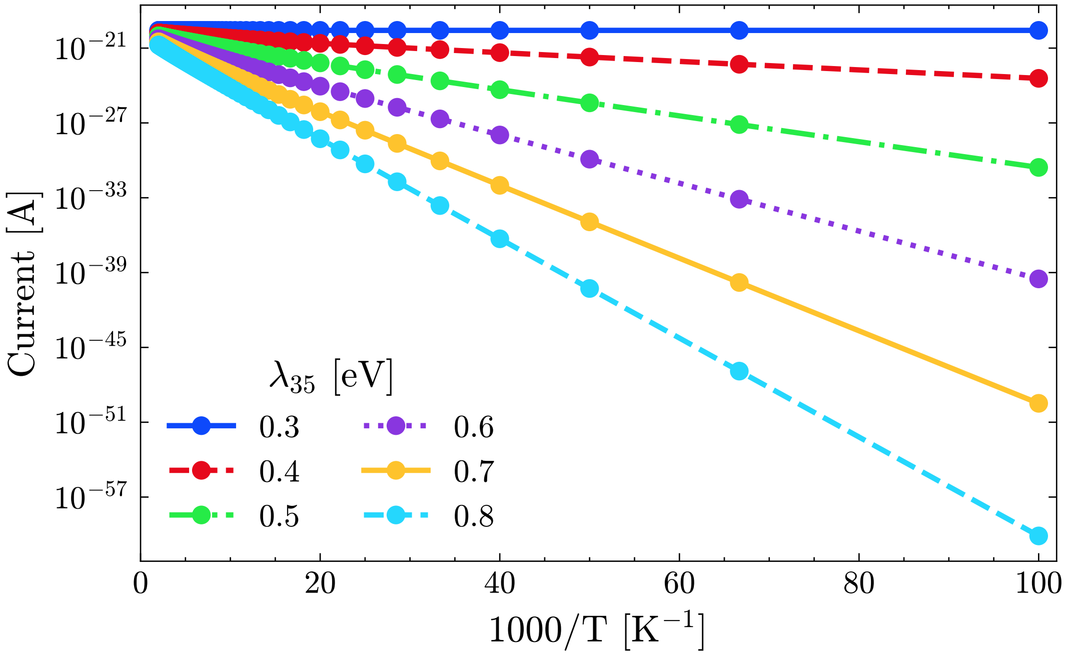

Figure S5: Dependency of the current on the temperature for different reorganization energies of residue HIS35. Data were extracted from the blinking scheme at $t = 466.6\,\text{ns}$ in the LUMO-pinned case.
